# Genetic background of neurological disorders with basal ganglia calcification

**DOI:** 10.1007/s00415-025-13344-1

**Published:** 2025-09-14

**Authors:** Maha Yektay Farahmand, Joel Wallenius, Johan Wasselius, Olof Gråhamn, Andreas Puschmann, Andreea Ilinca

**Affiliations:** 1 Division of Neurology, Department for Clinical Sciences Lund, Lund University, Lund, Sweden; 2 Department of Neurology, Skåne University Hospital, Inga Marie Nilssons Gata 47, 205 02 Malmö, Sweden; 3 Department of Neurology, Skåne University Hospital, Lund, Sweden; 4 Department of Radiology, Skåne University Hospital, Lund, Sweden; 5 Stroke Imaging Research Group, Department of Clinical Sciences, Lund University, Lund, Sweden; 6 Science for Life Laboratory, Department for Clinical Sciences Lund, Lund University, Lund, Sweden

**Keywords:** Basal ganglia calcifications, White matter hyperintensities, Whole genome sequencing, Monogenic neurological disorders, Stroke

## Abstract

**Background:**

Bilateral basal ganglia calcifications (BGCs), if severe, are known hallmarks for idiopathic BGC disease (IBGC), but if milder, are often considered radiological findings of unknown significance. In previous studies, only a minority of patients with BGC had monogenic forms of IBGC.

**Methods:**

We studied consecutive patients from a tertiary neurology clinic with bilateral BGCs of variable severity, and their families. We analyzed known IBGC genes, and an extended panel of genes linked to monogenic stroke and metabolic conditions. Clinical, radiological, and genetic data were collected, including vascular risk factors, cerebrovascular events, imaging findings (total calcification score, white matter hyperintensities, ischemic/hemorrhagic lesions), and relevant family history.

**Results:**

Twenty-four families with BGCs and neurological symptoms were analyzed. Disease-causing variants were identified in 14 families (58.3%). Eight patients had IBGC (variants in *SLC20A2, PDGFB, MYORG*), 4 had mitochondrial disease (*MT-TL1*), and 2 had monogenic vascular conditions (*GAL*, *MAP3K6*). Three variants were novel. BGC severity was highest in IBGC cases, while vascular and mitochondrial cases had milder calcifications. White matter hyperintensities were seen in 94.7% of cases and correlated highly with the total calcification score. Clinical vascular events had occurred in 41.7% cases. No monogenic cause was found in 10 patients, although many of these showed clinical or radiological features suggestive of monogenic disease.

**Conclusions:**

Bilateral BGCs can occur in many neurogenetic disorders apart from IBGCs, and a broader genetic search increases the diagnostic yield. Patients with BGCs frequently had clinical cerebrovascular events, which emphasizes the role of cerebrovascular pathology in BGCs.

## Introduction

Bilateral calcifications of the basal ganglia (BGCs), with or without calcifications in other brain regions, are the hallmark of rare monogenic diseases collectively called idiopathic basal ganglia calcification (IBGC; Fahr disease) [1]. Clinically, these disorders are characterized by neurological and psychiatric disease signs or symptoms, with a wide range of phenotypic expression and with reduced clinical penetrance [2]. Pathogenic variants in 8 different genes are currently known to cause IBGC [3]. Case series analyzed for IBGC genes have identified a monogenic cause in 19–47% of patients and families with BGCs [4, 5].

While BGCs can also be caused by endocrine or infectious diseases, or associate with aging [6], BGCs of variable severity may occur in other neurogenetic disorders [7], and both genetic and mechanistic studies have highlighted an association of BGCs with MRI white matter hyperintensities and with cerebral small vessel disease and/or dysfunction of small vessels [8, 9].

Here, we analyzed a series of 24 consecutive patients with BGCs and their relatives (genetic data from 38 and clinical data from 68 patients) from a tertiary neurology center for disease causes in known IBCG genes and in a large number of additional genes. These included genes associated with monogenic stroke or with white matter disease that had previously been associated with basal ganglia calcifications [7, 10]. Our aim was to determine the diagnostic yield of such comprehensive genetic analyses for determining the genetic cause of BGC patients and families. Furthermore, we described the clinical, radiological and genetic parameters in the various disorders with BGC in our series.

## Methods

Thirty-nine index patients aged 18 or older who had contact with our department’s neurology outpatient clinics in Lund or Malmö because of neurological signs or symptoms in 2022–2024 and who had bilateral symmetric calcifications in the basal ganglia were identified. Fifteen patients either did not wish to enroll in the study or to be genetically investigated. All enrolled patients who did not have a genetic cause for their disease determined by clinical genetic testing at the start of our study underwent whole exome or whole genome sequencing (WES, WGS) within the study. All 24 enrolled patients gave written consent to participate in the study. The study was approved by the Swedish Ethical Review Authority.

Clinical signs or symptoms previously related to idiopathic BGC (IBGC), including psychiatric, cognitive, movement disorder, seizure, headaches [11] and cerebrovascular disease manifested as transitory ischemic attack (TIA), stroke, ischemic or hemorrhagic lesions were documented. Radiological and genetic data were compiled. All index patients were interviewed and examined by one of the neurologist authors (A.I., O.G., A.P., M.Y.F.). We recorded age at onset of the first symptoms that were attributable to the underlying cause, as well as sex, clinical manifestations, comorbidities and family history. We included the index patients’ affected relatives for clinical radiological and genetical studies whenever this was possible. We also evaluated the presence or absence of clinical and/or radiological signs and symptoms of cerebrovascular disease, and classical vascular risk factors (hypertension, diabetes, hyperlipidemia, ischemic heart disease, smoking). The presence of vascular events was defined as a history of TIA, stroke, or cerebral bleedings or radiological signs of ischemic brain lesions. All index patients had normal parathyroid hormone, calcium, magnesium and vitamin D3 levels. None had a history of perinatal infections or trauma, nor uncorrected thyroid pathologies.

The size and intensity of intracerebral calcifications in CT-scans were classified using a previously published visual rating system that grades calcifications from 0 to 5 (0, absent calcification; 5, severe and confluent calcification) in 16 predefined anatomical locations and then determines the Total Calcification Score (TCS) as the sum of these (maximum 80) [1]. The extent of white matter hyperintensities (WMH) in the entire brain was rated as 0 = absent, 1 = punctate foci, 2 = beginning confluence, 3 = large confluent areas, based on the Fazekas scale for deep white matter lesions [12]), on MRI if available, otherwise CT hypodensities were documented. The presence of ischemic brain lesions or hemorrhages, as well as other relevant neuroradiological findings, was also noted. We examined the correlation of WMH in MRI with the patient’s TCS scores from CT images, using Kendall’s tau test.

WES or WGS data was analyzed; apart from the known monogenic causes for IBGC (monogenic IBGC: *CMPK2, JAM2, MYORG*, *NAA60, PDGFB*, *PDGFRB*, *SLC20A2*, and *XPRI*), patients were genetically screened for monogenic stroke conditions associated with BGC (*ACP5*, *ADA2*, *COL4AI*, *COL4A2*, *COLGALT1*, *CSF1IR*, *CTC1*, *FOXC1I*, *GJA1*, *GLA*, *JAM3*, *KRIT1*, *MAP3K6*, *PTEN*, *SAMHD1*, *SNORD118*, *STAT2*, *STING1I*, *TSC2*, *TTR*, *USP18*) from our stroke gene panels [10, 13] and for metabolic genetic conditions known to associate with bilateral BGC (variants in *ABCD1*, *CA2*, *CASR*, *CYP27A1, CYP2U1*, *DHFR*, *FA2H*, *FOLR1*, *GALC*, *GCM2*, *GNAI1*, *GNAS*, *PDE4D*, *PDE3A*, *PRKAR1A*, *PTH*, *SLC46A1*, *SOX3*, *TREM2*, *TYROBP*, as well variants in the mitochondrial DNA)[7]. BAM files were re-mapped to hg38 using Samtools [14] and bwa-mem2 [15]. Variant calling was performed with GATK [16] in regions defined by the gene panel’s transcript coordinates. VEP [17] was then used to annotate and filter the variants. Variants with minor allele frequencies < 1%, and Combined Annotation Dependent Depletion score > 25 were identified, classified according to the American College of Medical Genetics recommendations and clinically validated. In-house scripts were used to facilitate an overview of the results. IGV [18] was used to validate potential findings.

## Results

**Fig. 1:**
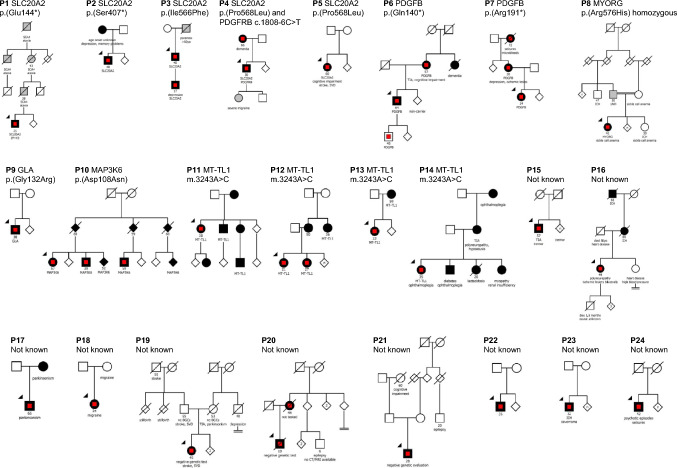
**Pedigree drawings of the 24 families with BGC described in this work.** Black symbols indicate individuals with a clinical manifestation ascribed to the underlying genetic cause (in the families of P1–P14) or by signs and symptoms compatible with known diseases with BGC (in the families of P15–P14). Grey symbols indicate clinical manifestations of unclear significance, red squares indicate persons with bilateral basal ganglia calcifications. Arrowheads indicate probands. Double line (P8) indicates consanguinity. Figures beneath symbols denote age (years) at first symptom. TIA, transitory ischemic attack; SCA4, spinocerebellar ataxia 4; SVD, small vessel disease. Data on P1, P6, P7 and P10 have previously been published [9, 19, 20]

Twenty-four index patients were identified, of whom 41.6% were females. Their clinical characteristics, including symptoms at onset, main neurological symptom, vascular events and the presence or absence of classical vascular risk factors are reported in Table 1. Their ages of onset were from 14 to 65 (median 38) years. We have compiled clinical data on 44 family members of the 24 index patients (Fig. 1).

**Table 1:** Clinical and genetic data of the 24 probands

P	Gene/variant	ACMG	AD/AR	Symptoms at onset (age)	Primary neurological symptom presumably related to BGC	Intracerebral calcifications (TCS, 0–80)	Number of bilateral locations with severe and confluent calcification (calcification score 5)	WMH intensity (0–3)	VRF
1	Heterozygous SLC20A2 c.1240 G > T p.(Glu144*)[19]	LP, novel	AD	Cerebellar ataxia (SCA4, not related to BGCs) (25y)	Facial twitches	10	1	No hypodensities in the white matter (CT)	0
2	Heterozygous SLC20A2 c.1220C > A p.(Ser407*)	LP, novel	AD	Seizure (48y)	Personality changes, memory problems	46	3	2 (MRI)	0
3	Heterozygous SLC20A2 c.1696A > T p.(Ile566Phe)	VUS^a^, novel	AD	TIA (40y)	Cognitive impairment	60	6	3 (MRI)	0
4	Heterozygous SLC20A2 c.1703C > T p.(Pro568Leu) and heterozygous PDGFRB c.1808-6C > T	P/LP and VUS	AD	Anxiety (30y)	Hearing loss, anxiety, mild personality change	40	3	No hypodensities in the white matter (CT)	0
5	Heterozygous SLC20A2 c.1703C > T p.(Pro568Leu)	P/LP	AD	Mild cognitive impairment (60y)	Stroke (67y)	8	0	3 (MRI)	1
6	Heterozygous PDGFB c.571C > T p.(Arg191*)[9]	LP	AD	Depression, Anxiety (14y)	Absence, seizure, TIAs (25y)	4	0	2 (MRI)	0
7	Heterozygous PDGFB c.418C > T p.(Gln140*)[9]	LP	AD	Balance difficulties (64y)	Balance difficulties	46	4	3 (MRI)	0
8	Homozygous MYORG c.1727G > A p.(Arg576His)	LP	AR	Gait/balance disturbance (41y)	Walking and speech difficulties	73	7	3 (MRI)	0
9	Heterozygous MAP3K6 c.322G > A p.(Asp108Asn)[20]	LP	AD	Mild cognitive impairment (57y)	Mild cognitive impairment	4	0	2 (MRI)	1
10	Heterozygous GLA c.394G > C p.(Gly132Arg)	P	AD	Gastric pain episodes (38y)	Stroke (47y)	14	0	2 (MRI)	0
11	MT-TL1 m.3243A > C	P	Mit	Headaches/migraine (38y)	Headaches	6	0	0 (MRI)	2
12	MT-TL1 m.3243A > C	P	Mit	Derealization, headaches (21y)	Headaches, depression episodes	12	0	1 (MRI)	1
13	MT-TL1 m.3243A > C	P	Mit	Renal insufficiency and uncontrolled hypertension (23y)	Cognitive impairment	20	1	2 (MRI)	4
14	MT-TL1 m.3243A > C	P	Mit	Ophthalmoplegia (35y)	Myopathy and fatigue	8	0	1 (MRI)	0
15	Negative	Nd	Sporadic	TIA (57y)	Tremor	4	0	1 (MRI)	3
16	Negative	Nd	Sporadic	Motor polyneuropathy (40y)	Motor polyneuropathy	2	0	Hypodensities in the white matter compatible with Fazekas 3 (CT)	1
17	Negative	Nd	AD	Parkinsonism (65y)	Parkinsonism	4	0	Hypodensities in the white matter compatible with Fazekas 1 (CT)	2
18	Negative	Nd	Sporadic	Headaches (24y)	Headaches/Parkinsonism	4	0	No hypodensities in the white matter (CT)	0
19	Negative	Nd	Sporadic	Essential tremor (45y)	Stroke (54y)	48	4	2 (MRI)	0
20	Negative	Nd	Sporadic	Seizures (19y)	Walking difficulties	34	2	2 (MRI)	0
21	Negative	Nd	Sporadic	Seizures (28y)	Seizures	4	0	1 (MRI)	0
22	Negative	Nd	Sporadic	Balance disturbance (35y)	Balance difficulties, speech difficulties	58	4	2 (MRI)	0
23	Negative	Nd	Sporadic	Balance disturbance, speech difficulties (32y)	Balance difficulties, speech difficulties	2	0	1 (MRI)	0
24	Negative	Nd	Sporadic	Psychotic episodes (42y)	Seizures	4	0	1 (MRI)	0

*ACMG* American college of medical genetics, *AD* autosomal dominant, *AR* autosomal recessive, *BGC* basal ganglia calcifications, *Nd* not determined, *LP* likely pathogenic, *Mit* mitochondrial, *P* pathogenic, *TCS* total calcification score, *TIA* transitory ischemic attack, *VUS* variant of unknown significance, *WMH* white matter hyperintensities, *VRF* vascular risk factors: hypertension; diabetes; hyperlipidemia; heart disease or smoking, *y* years of age

^a^same variant also identified in son who had BGC and neurological symptoms, see Case Vignette in Results section

### Genetic findings

Genetic investigations using WGS for 20 index patients, WES for 2 patients, NGS panel examination and Sanger sequencing (for one patient each) identified pathogenic and likely pathogenic variants in 13 index patients and one variant of unknown significance in 1 patient and his affected son (Table 1). Genetic data of 14 relatives of the index patients were evaluated to assess for co-segregation of the clinical and radiological phenotype with the genetic findings (Fig. 1). Of the 24 index patients, 8 had monogenic IBGC (variants in *SLC20A2* in 5 cases, *MYORG* in 1 case, *PDGFB* in 2 cases), 4 had a mitochondrial disease phenotype and *MT-TL1* m.3243A > C variants, and 2 had monogenic stroke conditions related to *MAP3K6* and *GAL*. Three of the detected variants were novel (Table 1). In several families, affected relatives carried the same variants as the index patients (Fig. 1).

### Vascular manifestations

Vascular disease manifestations were found in 9 (37.5%) of the 24 index patients: Stroke (3 cases), TIA (4 cases) and asymptomatic bilateral lacunar ischemic lesions (1 case) were noted in 8/24 (33% of index patients), cerebral bleeding related to cavernoma (1 case), and an asymptomatic splenic arterial aneurysm was detected (in 1 case who also had had TIA). A majority, 17 (68%) of 24 patients, did not have any classical vascular risk factor at the onset of neurological symptoms. Of the 8 index patients with monogenic IBGC, 3 (37.5%) had vascular disease manifestations (lacunary TIA syndrome at 25, respectively, 40 years of age, lacunary stroke syndrome at 76 years). One of the two patients with monogenic stroke conditions had vascular events (cardioembolic stroke at 38 years in the patient with Fabry disease).

### Intracranial calcifications

Brain CT images of all 24 index patients, obtained at a median age of 47 (range 5–76) years were re-evaluated. The 8 index patients with monogenic IBGCs had high radiological calcification TCS (mean 35.9, Table 1). However, TCS, as well as the number of bilateral locations with severe and confluent calcifications (visual grade 5—“severe and confluent” per location), varied widely between individuals within this group, between those with variants in the same gene, and even carriers of the same variant (Table 1). By contrast, the patients with monogenic stroke conditions (Fabry disease and *MAP3K6*-related disease) had “faint” to “moderate” calcifications in individual locations, and lower TCS (4 and 14), whereas the patients with mitochondrial disease had calcifications ranging from “faint” to “severe and confluent” in individual locations and a mean TCS of 11.5.

### White matter changes

**Fig. 2:**
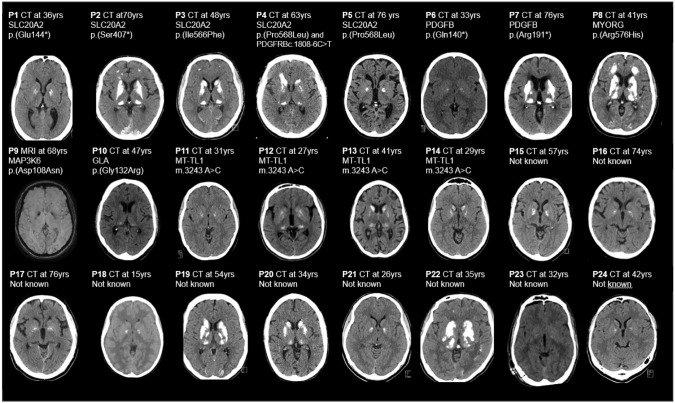
**Neuroradiological images of the 24 index patients**

MRI brain images were available for 19 index patients (Table 1). Of the 8 index patients with diagnosed IBGC, six (75%) had confluent WMH in a pattern compatible with cerebral small vessel disease. Both patients with monogenic stroke conditions had moderate WMH. The patients with mitochondrial disease without stroke-like episodes (P11, P12, P14) had absent to discrete, presumably nonvascular, WMH; one MT-TL1 m.3243A > C carrier who had had stroke-like episodes had moderate WMH (P13) (Fig. 2).

**Fig. 3:**
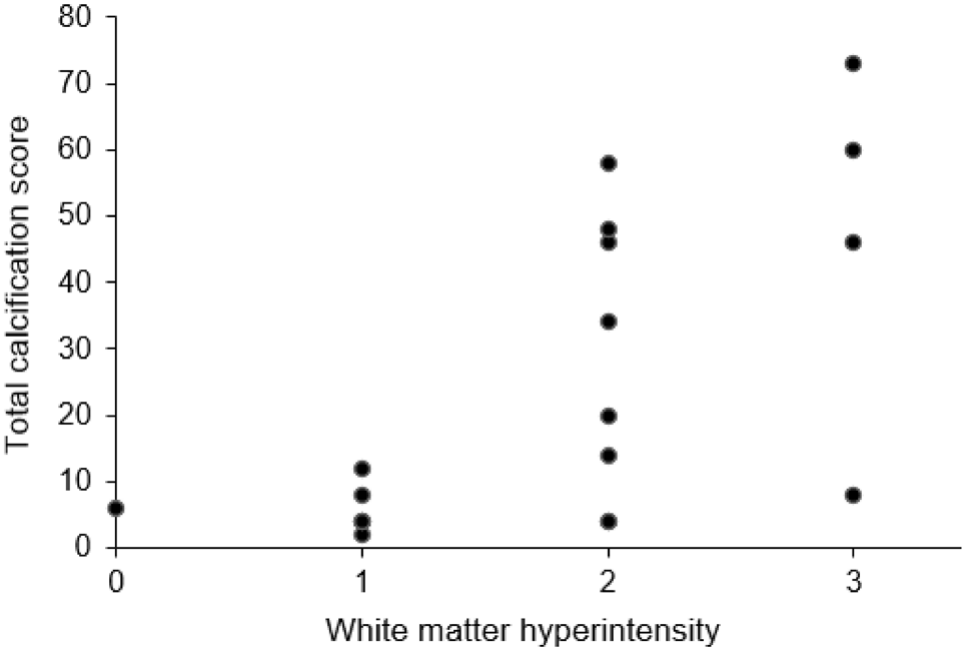
**Correlation of white matter hyperintensity with intracerebral calcification.** Each dot represents one of the 19 patients for whom MRI images were available. Total calcification score for each of them is shown

The degree of WMH showed medium to high correlation to the TCS (Kendall’s tau = 0.55), and the correlation was significant (*p* = 0.0041, Fig. 3). We observed no correlation of TCS with the age of the patient at examination in all 24 index patients (Kendall’s tau = 0.11, *p* = 0.4803) nor between patients with variants in the same genes (Fig. 2).

### Clinical case vignettes

This paragraph provides the clinical characteristics of index patients with monogenic causes for BGC, and their family members.

### SLC20A2

Four index patients with IBGCs were diagnosed with putatively pathogenic variants in *SLC20A2*. Index patient **P1** carried a truncating variant at c.1240G > T; p.(Glu144*) that had not previously been described, but was classified as likely pathogenic as other *SLC20A2* variants generating a premature stop-codon are known to cause disease. While IBGCs’ clinical spectrum includes cerebellar ataxia [1], the presence of similar cerebellar symptoms and sensory neuropathy with areflexia in other family members without BGCs made us to continue searching for other causes and lead to the identification.

of a GGC (poly-glycine) repeat expansion in the final exon of *ZFHX3* compatible with SCA4 [19]. We thus concluded that the balance disturbance, atactic speech, swallowing difficulties and the sensory polyneuropathy with areflexia co-segregating in the family were caused by SCA4, while possibly his rapid involuntary facial twitches, not seen in any other family member, may be explained by the *SLC20A2*-related condition.

**P2** had an unexplained seizure at 42 years. Personality changes and memory problems were gradually noticed why the patient was referred to a neurologist. He carries a novel likely pathogenic truncating variant *SLC20A2* c.1220C > A; p.(Ser407*). His mother was diagnosed with depression and memory problems, as well as radiologically with BGCs (Fig. 2).

**P3** carried *SLC20A2* c.1696A > T; p.(Ile566Phe), not previously described, which was assessed as deleterious in silico and was also identified in his symptomatic son (Fig. 1). P3 had mild psychiatric symptoms with disinhibition, depression, personality change, irritability, memory problems with signs of frontal and temporal lobe deficits on cognitive testing. He displayed mild chorea. His son had bilateral BGC, a clinical diagnosis of attention deficit hyperactivity disorder, depression with repeated suicidal attempts, and anxiety, and reported infrequent episodes of involuntary movements when treated with atomoxetine. Both P3 and his son had hearing loss.

Patient **P4** carried a heterozygous very rare intronic variant in *PDGFRB* c.1808-6C > T of unclear significance and a likely pathogenic heterozygous variant in *SLC20A2* c.1703C > T; p.(Pro568Leu) that had previously been described [21–23]. BGCs were identified on a brain CT that was performed to evaluate a sinusitis. However, his history revealed hearing loss, vitreous detachment, autoimmune thyroiditis necessitating substitution with levothyroxine, and mild anxiety and personality self-perceived personality changes. His mother developed cognitive decline at 66 years and received a diagnosis of dementia in her 90 s, she had severe BGCs and bilateral cerebellar calcifications.

**P5** carries the same rare *SLC20A2* variant as P4. The patients are not related to each other. P5 reported slowly progressive subjective cognitive impairment starting at 60 years, and a stroke episode at 67 years of age that was classified as related to small artery occlusion. She had a diagnosis of hypertension at the time of the stroke episode, which was well-treated with one antihypertensive. No heredity for clinical signs of IBGC was reported and no radiological investigations of her relatives were available.

Of the 5 index patients with *SLC20A2* variants, 2 (P3 and P5) had had vascular events—TIA at a young age in P3 and stroke at 67 years in P5—and both also had confluent WMH (grade 3). The intracerebral calcifications in all five patients with pathogenic *SLC20A2* variants were moderate (TCS3) to severe and confluent (TCS5, Table 1) with TCS from 8 to 60/80. All five *SLC20A2* carriers reported other family members with the same condition in a pattern compatible with an autosomal dominant model of inheritance.

### PDGFB

Index-patients **P6** and **P7** with truncating, likely pathogenic variants in *PDGFB* were previously reported [9]. They manifested clinically with both degenerative and vascular phenotypes with an autosomal dominant mode of inheritance with incomplete penetrance. Radiologically, calcifications with a variable TCS of 4 and 46/80 and confluent WMHs (grade 2 and 3) were detected.

### MYORG

**P8** was homozygous for *MYORG* c.1727G > A; p.(Arg576His). At 41 years, the patient presented to our clinic and reported that she had had balance problems, dysarthria and weakness of the right arm and right side of the face. Stroke was initially suspected, but her symptoms gradually deteriorated in the subsequent weeks, and after an additional month spontaneously improved. Brain CT, CT-angiography and MRI excluded acute vascular lesions but revealed confluent (grade 3) WMHs and intensive and widespread intracerebral calcifications (TCS 73). Extensive workup for markers of inflammation or vasculitis was negative. Lumbar puncture showed normal cell count and albumin levels, but markedly elevated concentration of neurofilament light chain in the cerebrovascular fluid (13,500 ng/L; laboratory reference < 632). She was known to have sickle-cell anemia. She reported that three family members had strokes at early ages; her sister had an intracerebral hemorrhage at age 30, her father ischemic stroke at 50, her paternal uncle intracerebral hemorrhage at 47) but there was no family history of cognitive, psychiatric or movement disorder signs or symptoms. P8’s parents were cousins.

### GLA

**P9** developed episodes of gastric pain at 38 years of age and had cardioembolic stroke at 47 years. He carried the pathogenic *GLA* c.394G > C; p.(Gly132Arg) variant. He also developed cardiomyopathy, scleritis, Raynaud phenomenon, deep vein thrombosis, and lung embolism, as well as hypoacusis. No other family member had vascular diseases or degenerative diseases. His intracranial calcifications were faint (14/80 TCS) and most visible in the thalami, and he had discrete WMH of presumably vascular origin. A diagnosis of Fabry disease was confirmed by biochemical analyses after his stroke episode.

### MAP3K6

**P10** had mild cognitive complaints that progressed very slowly, during the course of a few years, and with episodes of dizziness related to low blood pressure when she was standing erect. She carried a *MAP3K6 c.322G* > A; p.(Asp108Asn) that co-segregated with a cerebrovascular phenotype in a larger family, as previously described [20]. Three affected and no unaffected family members presented faint BGCs and discrete WMHs [20], suggesting BGCs as a possible disease trait with incomplete penetrance.

### MT-TL1

Four index patients (**P11**, **P12**, **P13**, **P14**) with bilateral BGCs who carried the MT-TL1 m.3243A > C variant were addressed to our neurology clinic because of headache episodes, cognitive impairment, or muscular fatigue. These symptoms had started at ages 21–38. None of them had had vascular events or stroke, and three had already been diagnosed with at least one classical vascular risk factor at the moment of our evaluation. Their intracranial calcifications were moderate, with TCS between 6 and 20 points, and they had absent to moderate WMH, grade 0 to 2. One of them, P13, who also developed stroke-like episodes, had moderate cerebellar and discrete parieto-frontal atrophy bilaterally. All the index patients had at least two other affected family members, with a maternal inheritance pattern (Fig. 1).

### Patients with no genetic diagnosis

For 10 (42%) patients, no monogenic disease cause was identified. Of these, only 3 had “severe and confluent” calcifications in any intracranial location, with high TCS values of 34, 48 and 58, which might suggest an IBGC-related condition. The remaining 7 had low TCSs of 2 or 4. Four of these 10 index patients with negative genetic workup had vascular disease manifestations (TIA, ischemic stroke, bilateral ischemic lesions, intracerebral bleeding caused by cavernomas), and two of them reported heredity for cerebrovascular disease (stroke, TIA, ischemic lesions, intracerebral bleeding; P16 and P19). Family history for similar clinical manifestations as in the index patient (tremor, epilepsy, psychiatric disease, stroke) was positive in 5 of these 10 families in a pattern compatible with a monogenic cause (Fig. 1).

## Discussion

We studied 24 index patients and families with neurological symptoms and bilateral BGCs of different volumes and intensities. Analyses of 50 genes identified a monogenic causative condition in 14 (58.3%) index cases and families; 8 associated with IBGC conditions, 2 with monogenic stroke conditions and 4 with mitochondrial disease.

A difference between the present study and previous publications on BGC-associated monogenic diseases [2, 4, 5, 24] is that we also included index patients with milder calcifications, and that we performed broader genetic screening of a total of 50 nuclear genes and of mitochondrial DNA, including not only IBGC-genes but also genes for monogenic stroke conditions and for genetic metabolic conditions known to associate with bilateral BGC [3, 4, 7].

Previous studies reported that 19% [4], 20% [2], 44% [24] or 47% [5] of index patients with BGC carried pathogenic or likely pathogenic variants in genes related to IBGC. Although our study included those with relatively mild basal ganglia calcifications, where the monogenic primary forms are considered less likely, our approach provided a diagnosis of a monogenic disorder for a larger proportion (58.3%) of index patients and their families. We found that some of our patients with milder BGC had genetic conditions other than the known genetic forms of IBGC.

Furthermore, we found that almost all patients with BGC have white matter changes, considered to be related to either small vessel disease or to mitochondrial dysfunction. There was a strong statistical correlation between the presence or extent of WMH and intracerebral calcification load, as assessed with the well-established TCS. This confirms similar findings from previous studies [8, 25–27]. In addition, our study showed that not only radiological but also clinical manifestations of cerebrovascular disease, including previous ischemic or hemorrhagic stroke, and TIA, were common among the patients studied here and their affected relatives. This emphasizes a pathomechanistic connection between abnormalities in small blood vessels or in cellular energy metabolism with intracerebral calcifications in patients with neurological symptoms [27–29]. In contrast to previous studies [6], we did not observe any correlation of patient age and intracerebral calcification load. We observed the highest TCS values in patients with pathogenic variants in the known IBGC genes (*SLC20A2*, *PDGFB*, *MYORG*), but also noted that three patients (P19, P20, P22), in whom we did not find any genetic causes, had severe calcifications. Furthermore, we observed a wide range of TCS values from 4 to 60 among patients with *SLC20A2*- and *PDGFB-*related disease*.* By contrast, the patients with *MT-TL1, MAP3K6,* and *GLA* variants only had milder calcifications (TCS 4–20).

The following mechanisms have been identified or suggested for the causative genes reported in this work: *SLC20A2* encodes a sodium-phosphate transporter and pathogenic variants in this gene may cause intracellular ion accumulation and deposition [30]. Pathogenic variants in *PDGFB* cause deficiency of pericyte function and of blood–brain barrier integrity [31]. *MYORG* is highly expressed in astrocytes, and the pathomechanism for recessive *MYORG*-related BGC may be a loss of function of the neurovascular unit, in turn affecting the blood–brain barrier [32, 33]. In *MAP3K6*-related disease, there is *post-mortem* evidence for degeneration of the muscular lamina interna and the elastic layers of basal ganglia arteries [20]. The pathophysiology of disease caused by *MT-TL1* m.3243A > C implies mitochondrial dysfunction that may cause mitochondrial angiopathy and/or degenerative changes [34, 35]. In Fabry disease, pathological accumulation of globotriaosylceramide in smooth and endothelial muscle cells leads to an impairment of blood vessels [36]. For many of the other genes on our gene list, the exact pathomechanism that leads to BGC may remain unknown. At the same time, this variability of pathophysiological mechanisms mentioned above underlines that a wider range of molecular disturbances can converge towards the occurrence of BGCs.

Our study confirms that in a proportion (41.7%) of patients and families with neurological signs or symptoms and intracerebral calcification, with and without positive family history, no monogenic cause is presently identified. Various reasons may explain this. Short-read based sequencing may be unable to find certain types of genetic variants in known disease-associated genes; the disease cause may be in other genes or genomic areas outside the genes in our gene list. We assume that additional monogenic causes for IBCG will still be discovered in the future. We have not examined possible environmental or additional endocrinological disease causes [3], but during our careful re-evaluation found no suggestions of such causes in any of these individuals or families.

A key limitation of our study is the non-systematic selection of patients, as only those referred to our outpatient clinic were informed and considered for participation. Consequently, individuals assessed exclusively in our acute or inpatient departments were not included. However, this is a single-center study, which ensured that all included participants underwent consistent clinical, radiological, and genetic evaluations. A notable strength of the study lies in the comprehensive genetic analysis methodology employed, which enabled the exploration of potential associations between IBGC, varying in volume and intensity, and a large number of monogenic diseases. Additionally, this approach allows for the identification of possible links with other, albeit rare, but potentially actionable neurometabolic conditions, such as neurodegeneration due to cerebral folate transport deficiency, galactocerebrosidase deficiency, and cerebrotendinous xanthomatosis.

## Conclusions

We suggest that the presence of milder BGCs should also be included as relevant clinical information to direct the diagnostic workup of patients who present with neurological disease. Genetic testing of a broad range of monogenic disorders, for example, with the list of genes used in our work, can be indicated in patients with BCGs. Identification of the exact monogenic disease can have direct therapeutic consequences as exemplified by the 5 (20.8%) of 24 patients with *GLA* or *MT-TL1* m.3243A > C-related disease in this study. The connection between cerebrovascular disease mechanisms and BGCs warrants further study.

## Data Availability

The data that support the findings of this study are available from the corresponding author, [A.I.], upon reasonable request.
